# Mini-EmulsionFabricated Magnetic and Fluorescent Hybrid Janus Micro-Motors

**DOI:** 10.3390/mi9020083

**Published:** 2018-02-15

**Authors:** Jiapu Jiao, Dandan Xu, Yuhuan Liu, Weiwei Zhao, Jiaheng Zhang, Tingting Zheng, Huanhuan Feng, Xing Ma

**Affiliations:** 1 State Key Lab of Advanced Welding and Joining, Harbin Institute of Technology (Shenzhen), Shenzhen 518055, China; j15602980509@163.com (J.J.); xudandan@stu.hit.edu.cn (D.X.); wzhao@hit.edu.cn (W.Z.); zhangjiaheng@hit.edu.cn (J.Z.); 2 School of Materials Science and Engineering, Harbin Institute of Technology (Shenzhen), Shenzhen 518055, China; hitlyh182371@163.com; 3 Department of Ultrasound, Peking University Shenzhen Hospital, Shenzhen-PKU-HKUST Medical Center, Shenzhen 518036, China; kyzs_018@126.com; 4 Key Laboratory of Micro-systems and Micro-structures Manufacturing of Ministry of Education, Harbin Institute of Technology, Harbin 150001, China

**Keywords:** Janus particles, micro-motors, multiple functionalization, self-propelling, motion manipulation

## Abstract

Self-propelling micro/nano-motors have attracted great attention due to their controllable active motion and various functional attributes. To date, a variety of technologies have been reported for the fabrication of micro/nano-motors. However, there are still several challenges that need to be addressed. One of them is to endow micro/nano-motors with multi-functionalities by a facile fabrication process. Here, we present a universal approach, adopted from the emulsion templating method, for the fabrication of Janus micro-motors. With a one-step process, magnetic nanoparticles and fluorescent dyes are simultaneously embedded into the microparticles. The self-propelled motors can be used as an active label or fluorescent tracer through manipulation of their motion using magnetic guidance.

## 1. Introduction

Self-propelling motors are small autonomous devices which are capable of harvesting energy from their environment and mechanically driving themselves in fluids [1,2,3,4]. They have attracted great attention during the last decade due to their ability to drive and build intelligent micro/nano-robots that can accomplish designed tasks for different applications [5]. Although considerable challenges remain in order to move these micro/nano-motors towards practical real-world use [6], the promising future of this field continues to attract more and more scientists. Therefore, there have been a large number of researchers contributing great effort to fabricating various types of micro/nano-motors, and studying their motion behavior and potential applications during the last decade [3,4]. From the view of motor structure, researchers have reported on bimetallic nanorods [7,8], typical spherical Janus particles [9,10], bubble propelled tubular micro-jets [11,12,13,14], and magnetically driven micro/nano-helix, etc. [15,16,17]. Based on their special application or required functionality, different fabrication technologies, including electron beam deposition [7,8,11], supramolecular self-assembly [18], template-assisted electrochemical deposition [12,13], sol–gel chemistry [19,20,21,22], layer-by-layer assembly [14,23], and rolled-up method, etc. [24], have been utilized to produce micro/nano-motors. The demonstrated proof-of-concept applications include targeted cargo (drug) delivery [25,26] and imaging for biomedical purposes [27,28,29], pollutant degradation or remediation in environmental sedimentation [30,31,32], precise particles or cells’ manipulation [33,34,35,36,37,38], and biochemical sensing [39,40,41,42,43]. Aiming at those potential applications, the self-propelled micro/nano-motors should be controllable with regards to movement direction, as well as apparent labeling for motion tracking or potential in-situ imaging applications. 

For the manipulation of the motors’ movement, the most direct approach is to incorporate a magnetic element into the motors’ building structure and use an external magnetic field to guide the motors’ moving direction. Several examples were reported. First of all, the motors can be directly constructed with a magnetic component, such as the magnetic powered Helix, to achieve both magnetically driven self-propulsion and control of the motion direction [17,44,45,46,47]. Then, a common method is the use of magnetic motors via the deposition of a magnetic layer such as a ferromagnetic Ni, on the motors’ surface [48,49]. Baraban et al. reported progress on the magnetic guidance of spherical Janus particles [50]. They manage to achieve an alignment of the magnetic moment along the main symmetry axis of the spherical Janus micromotors by using a special magnetic cap structure based on ultrathin multilayers of [Co./Pt(Pd)]_N_. This specific composition exhibits a perpendicular magnetic anisotropy (magnetic moment points perpendicular to the sample surface), even when deposited on arrays of spherical particles with sizes ranging from 50 nm to 5 mm [51,52]. Samuel et. al. used a similar strategy to control the movement direction of mesoporous silica micro-motors for target cargo delivery as well [53]. For tubular micro/nano-jets, a common method is to electrochemically deposit a magnetic layer of Ni between the motors’ substrate and catalytic layers, so that the movement direction can be aligned by an external magnetic field [54]. Another strategy is to incorporate magnetic nanoparticles onto the motor’s body, by simple physical adsorption, which can not only provide magnetic properties to the motors but also make the motors capable of acting as a magnetic resonance imaging (MRI) tracer in biomedical use [55]. 

Additionally, the ability to fluorescently label or load micro/nano-motors molecules demonstrates both research and practical applications. For instance, fluorescent-labeled spheres were used to investigate the rotational behavior of active colloids [56]. Samuel et al. developed a series of mesoporous silica micro/nano-motors that could load and encapsulate fluorescent dyes or drug molecules for delivery and release [20,53,57]. Furthermore, the long-term scientific goal of the micro/nano-motors community is to utilize these motors for biomedical purposes, of which, the field of fluorescent imaging is one of the most common. Fluorescent imaging techniques are used for both in vitro and in vivo theranostic applications. Recently, Li Zhang et al. produced a biodegradable, fluorescent and magnetic helix micro-motor for in vivo imaging-guided drug delivery, which demonstrated the utility of producing motors with multiple capabilities [55]. Currently, a post-synthesis process of magnetic component incorporation, dye staining or fluorescent labeling is required to confer multiple functions on the motors. Thus, there are still challenges that require further development towards a new and facile strategy to fabricate micro/nano-motors with multiple functionalities, such as magnetism and fluorescence.

**Scheme 1:**
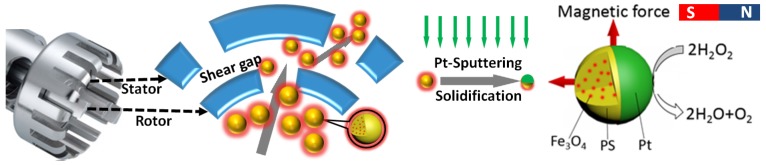
Scheme of magnetic/fluorescent hybrid micro-motors fabricated by mini-emulsion.

Here we present a universal approach adopted from the emulsion templating method for Janus micro-motors fabrication (see Scheme 1). By high speed stirring in a toluene and water mixture containing polystyrene (PS) polymers, we can obtain an oil/water emulsion containing large amounts of oil (toluene) micro-droplets in which the PS polymers were loaded. After evaporation of the toluene, the solid PS microparticles can be produced in a facile way. By adding hydrophobic magnetic nanoparticles and fluorescent dyes into the emulsion system, the PS microparticles or motors can be functionalized with both magnetism and fluorescence properties during one single emulsion process. This means that the motors can be used as an active biomedical label with motion manipulation via a magnetic field. By depositing a platinum (Pt) layer onto one side of the particles, we obtained Janus motors whose motion can be driven by asymmetric catalytic deposition of hydrogen peroxide (H_2_O_2_). We believe the multiple functionalized micro-motors hold great potential for varied applications, such as targeted cargo transportation, micro-manipulation, drug delivery, or future bio-imaging-guided micro–mini-scaled surgery.

## 2. Materials and Methods 

All materials were purchased from Sigma Aldrich (St. Louis, MI, USA) and used without any further purification.

### 2.1. Synthesis of Magnetic Particles Fe_3_O_4_

Magnetic nanoparticles (Fe_3_O_4_) were synthesized using the hydrothermal method. We used a modified method outlined in Dongyuan Zhao [58]. The procedure is described as follows. An amount of 0.675 g FeCl_3_·6H_2_O was dissolved into 35 mL glycol with constant sonication. An amount of 1.925 g CH_3_COONH_4_ was added into the solution and stirred for 30 min. The solution was loaded into a reactor and heated at 200 °C for 12 h to complete the reaction. Then, the samples were collected, washed by centrifugation, and air dried for future use. 

### 2.2. Synthesis of Magnetic Particles Fe_3_O_4_@SiO_2_

An amount of 40 mg of the previously produced magnetic nanoparticles are dispersed into a mixture solution containing 40 mL deionized (DI) water and 200 mL isopropanol and treated with sonication for 30 min. An amount of 7 mL ammonia (25 wt %) and 0.6 mL tetraethyl orthosilicate (TEOS) were added into the solution which was mechanically stirred for 4 h to complete silication. The samples were collected by a magnet and rinsed 3 times with ethanol.

### 2.3. Fabrication of Magnetic Particles Fe_3_O_4_@SiO_2_ with Fluorosilane Surface Modification

An amount of 50 mg of previously prepared magnetic Fe_3_O_4_@SiO_2_ nanoparticles were dispersed into 50 mL ethanol containing 0.5 mL fluorosilane. The solution was refluxed at 80 °C for 24 h. The samples were centrifuged and air dried for future use. The collected nanoparticles were deposited onto a glass slide for contact angle measurement. 

### 2.4. Fabrication of Magnetic and Fluorescent Polystyrene (PS) Microparticles

The micromotors were fabricated via a typical emulsion templating approach [59,60]. The protocol we used is as follows. An amount of 70 mg magnetic nanoparticles Fe_3_O_4_@SiO_2_ with fluorosilane surface modification and 0.1 g PS were dispersed into 10 mL toluene containing Nile Red dye (0.07 wt %) with sonication treatment for 30 min. The magnetic PS toluene solution was mixed with 70 mL sodium dodecyl sulfate (SDS) aqueous solution (10 mM concentration). The mixed solution was emulsified by Ultra Turrax T18 for 10 min with rotation speed 6000 rpm. The emulsion was mechanically stirred for 2 days to evaporate the toluene to produce solid magnetic PS microparticles.

### 2.5. Platinum Janus Polystyrene (PS) Microparticles

Magnetic PS microparticles were collected and placed on a glass slide to form a monolayer by drop coating. Then, the microparticles were coated with a thin layer of Pt by sputtering for 110 s at 10 mA current. Then, the obtained Janus motors were collected by sonication and suspended in DI water for further investigation.

## 3. Results and Discussion

**Figure 1:**
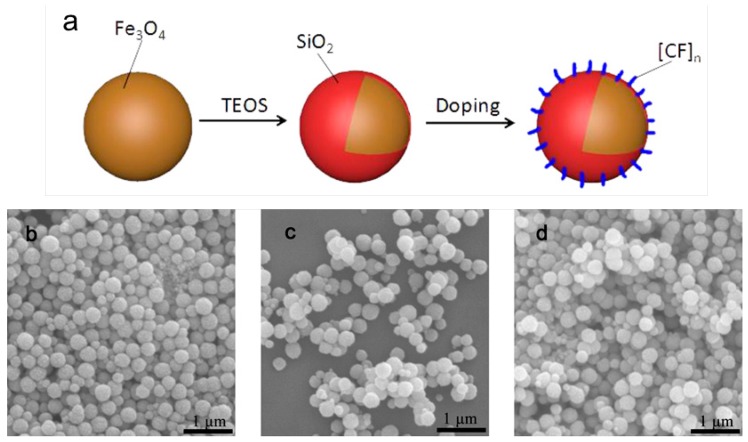
(**a**) Schematic illustration of the preparation hydrophobic magnetic nanoparticles and scanning electron microscopy (SEM) images of (**b**) the original magnetic nanoparticles; (**c**) after silica growth and (**d**) further modification with fluorosilane.

In order to have magnetic PS microparticles, we first synthesized magnetic nanoparticles (Fe_3_O_4_) and modified the surface properties, switching from hydrophilic, to hydrophobic as seen in Figure 1a. This conversion of surface property is necessary because during the emulsion process, instead of being suspended in the aqueous solution, the hydrophobic magnetic nanoparticles and water-insoluble fluorescent dyes prefer to be embedded in the hydrophobic toluene/PS spheres formed by high-speed shearing-induced emulsion. In other words, the magnetic nanoparticles need to stay in the oil phase (droplets) during the whole process. During further evaporation of toluene the solid PS microparticles loaded with both magnetic nanoparticles and fluorescent dyes will spontaneously form in the solution. 

### 3.1. Magnetic Particles Synthesis and Modification

As shown in Figure 1a, based on the magnetic nanoparticles we synthesized, a silica shell was grown on their surface through a modified Stöber reaction. Then, we continued surface modification via refluxing with perfluorodecyltrichlorosilane (fluorosilane) in ethanol solution to form a hydrophobic surface. The samples were characterized by scanning electron microscopy (SEM) as shown in Figure 1b–d. 

The original size of the magnetic nanoparticles was around 200 nm. The size increased to 300 nm due to the growth of the silica shell. The particles’ size did not change significantly after perfluorodecyltrichlorosilane modification (see Figure 1c) since the grafting polymer is quite short. The magnetic nanoparticles formed numerous aggregates after surface modification since their surface was modified to switch from hydrophilic to hydrophobic. The silica growth and surface modification were both verified by Energy-dispersive X-ray spectroscopy (EDS) analysis (see Figure S1). During every surface modification step, silicon element and fluoride appeared as expected. The surface modification is quite successful based on the contact angle measurement. The contact angle between water and the hydrophobic surface of the modified magnetic nanoparticles increased from 10 degrees to 165 degrees (the detailed measurements and result information are listed in Figure S2). The size of the Fe_3_O_4_ nanoparticles was quantified by dynamic light scattering (DLS) in origin, after silica growth, and after fluorosilane surface modification (results are presented in Figure S3). The size increased after silica shell growth due to actual size increasing, as well as slight aggregation as seen in the SEM image (Figure 1c). After surface grafting of fluorosilane, the size increased and was confirmed by DLS measurement, which may be due to aggregation of particles by hydrophobic interaction in aqueous solution. It is understandable that the particles’ surface shifting from hydrophilic to hydrophobic would lead to their aggregation in aqueous condition.

### 3.2. Magnetic/Fluorescent PS Microparticles Fabrication

Magnetic and fluorescent PS microparticles were fabricated through the emulsion templating method as described above. By using this method, the particles’ size can be tuned easily by adjusting the rotation speed of Ultra Turrax. We investigated the relationship between the particles’ size and rotation speed (results can be seen in Figure S4). In general, the particle size decreases exponentially with the increasing of the rotation speed. We have chosen an optimized condition at 6000 rpm for fabrication of micro beads, which results in an approximate particle size of 30 μm. Toluene evaporation during air drying resulted in smaller particles due to shrinkage attributed to volume loss. Then, we obtained the magnetic and fluorescent PS particles. The results of the fabrication process were characterized by SEM and EDX analysis (see Figure S5). We found iron in the formed magnetic microparticles (see Figure S5b).

**Figure 2:**
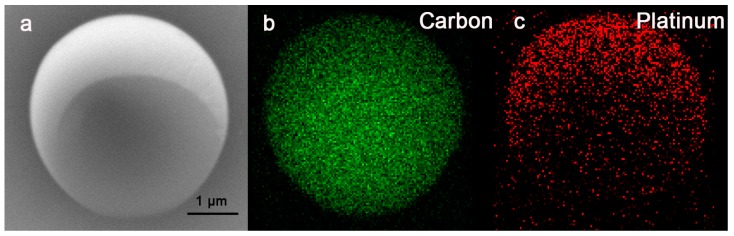
SEM photos of (**a**) magnetic polystyrene (PS) micro-particle, element mapping of (**b**) Carbon and (**c**) Platinum.

**Figure 3:**
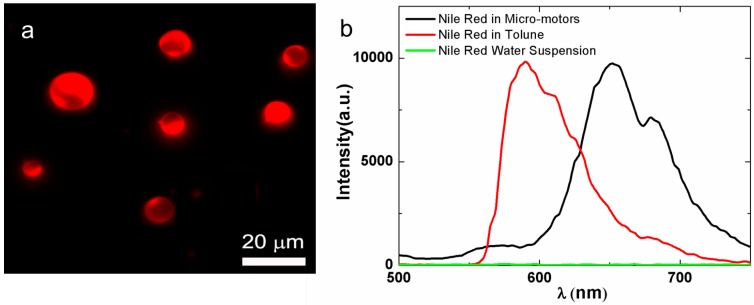
(**a**) Fluorescent microscopy image and (**b**) fluorescent emission spectrum of the magnetic/fluorescent hybrid micro-motors in aqueous solution.

The Janus motors were readily acquired by depositing a thin layer of Pt (10–20 nm) onto the PS particles by sputtering machine. Then, the finally obtained Janus magnetic and fluorescent micro/motors half coated with Pt were characterized by SEM and EDS (see Figure 2). SEM image analysis revealed a Pt shell covering the upper half of the PS particles (see figure 2a). EDS mapping also confirms the same element distribution of Janus shape (see Figure 2b,c. Green represents the presence of carbon and red represents the presence of Pt). We used fluorescent microscopy to observe the Janus motors and clearly observed red florescence emitted by Nile Red inside the motors (see Figure 3a). Then, we further measured the fluorescence spectrum of the micro-motors in aqueous solution (see Figure 3b). The spectrum confirmed the presence of Nile Red inside the micro-motors. Furthermore, although Nile Red possesses excellent optical properties, its hydrophobic characteristics and fluorescence emission would be quenched (see Figure 3b), which limits its biomedical application. However, in our strategy, we incorporated such water-insoluble dye into microparticles, thereby preserving their optical properties in aqueous environments. Therefore, we have successfully achieved the production of multifunctional, hybrid micro-motors which were equipped with both magnetic and fluorescent properties. Moreover, based on different applications or requirements, we can easily not only tune the concentration and thus emission strength of fluorescent dyes inside the motors, but also load different kinds of dyes (e.g., near infrared (NIR) for in vitro or in vivo imaging) based on the current strategy, which can greatly extend the utility of the fluorescent motors. 

### 3.3. Motion and Manipulation of the Micro-Motors

**Figure 4:**
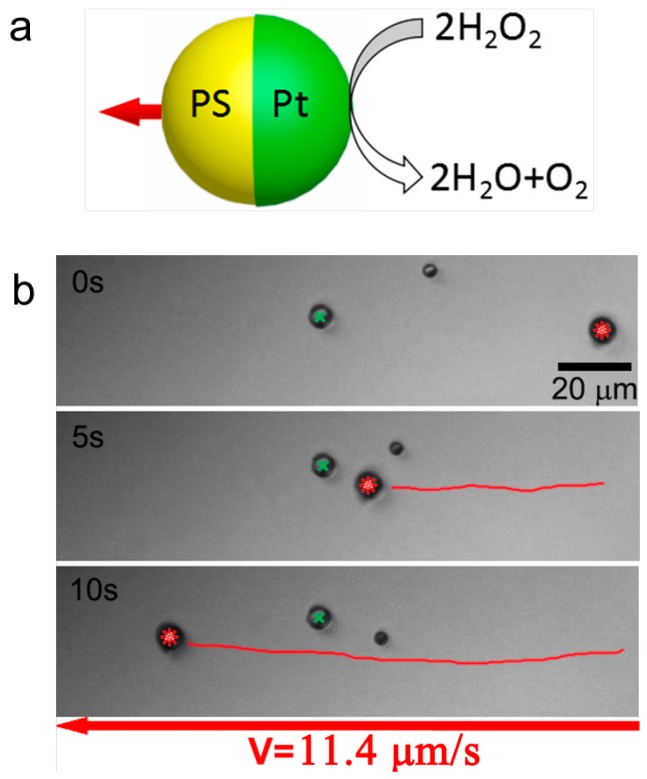
(**a**) Schematic illustration of the self-propelled Janus motors and (**b**) video snapshots of the motion of a micro-motor (red) with the addition of peroxide (3 wt %), without any surfactant. (The green particle stands as a reference particle.)

After obtaining the micro-motors, we studied their motion behavior by placing them into an aqueous solution fueled with H_2_O_2_ (3 wt %). A typical movement result is shown in Figure 4 together with a scheme illustrating the motion mechanism. The active motor was apparently self-propelled and moved from right to left as indicated by a red star mark. The green star marked passive spheres, which served as a position reference to clearly show the active motion of the Janus motors. The tracking trajectory was shown by a red line. The average velocity of this specific Janus motor was found to be 11.4 μm/s (RSD = 22.7%, *n* = 15), which agrees with other reported phoretic motors powered by Pt/H_2_O_2_ reaction. [3] The supporting video (Video S1) can be found in Section Supplementary Materials.

**Figure 5:**
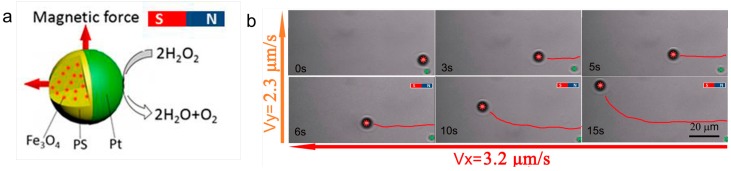
(**a**) Scheme and (**b**) video snapshots of a manipulated magnetic micro-motor with 3 wt % H_2_O_2_, no surfactant. The green particle stands as a reference particle.

The manipulation of the magnetic micro-motors is shown in Figure 5. The magnetic micro-motor was moving from right to left at the beginning without any magnetic force manipulation. It is clear that the motor was moving almost straightforward. Then, we applied a magnetic field by using a permanent magnetic bar. The magnetic field at the motors’ position was about 40 mT. After we applied a magnetic field from the perpendicular direction to give external guidance to the motors, we found an extra movement originating from the bottom side upwards which can be seen in the video (see Figure 5b). The motors showed corresponding motion after the magnetic force was applied. Due to the spherical shape and random distribution of magnetic nanoparticles inside the motors, no exact orientation was attributable to the magnetic moment. However, in our case, both magnetic attraction and orientation were working on the motors to change the moving direction, which works very well from our observation. The micro-motors’ moving direction changed from horizontal to vertical after application of the magnetic field. The manipulated motion can be projected onto X and Y—two directions to simplify the analysis. The speed of self-propulsion, as measured on the X axis, was 3.2 μm/s. The speed of magnetic force, as measured on the Y axis, was 2.3 μm/s (Video S2 can be seen in Section Supplementary Materials). 

## 4. Conclusions

We have presented a universal approach, adopted from the emulsion templating method, for the fabrication of Janus micro motors. The motors can be functionalized with both magnetism and fluorescence within a one-step process. Such self-propelled systems can move inside aqueous solutions when fueled with 3 wt% H_2_O_2_, and exhibit strong fluorescent emission in aqueous solution for potential applications in bio-imaging and particle-tracking, etc. Furthermore, due to the presence of magnetic nanoparticles inside the motors, their directionality can be easily manipulated by applying an external magnetic field, leading to controllable and multi-functional motors. Therefore, our motors not only provide a new strategy to fabricate magnetic/fluorescent motors in a facile way but also prove the potential of these motors for future applications, such as targeted cargo delivery and micro-manipulation.

## Supplementary Materials

The following are available online at http://www.mdpi.com/2072-666X/9/2/83/s1. Supplemental Figure S1: Energy-dispersive X-ray spectroscopy (EDS) of Fe_3_O_4_ (S1a), Fe_3_O_4_@SiO_2_ (S1b) and fluorosilane surface modification Fe_3_O_4_@SiO_2_ (S1c); Figure S2: Contact angle measurement of Fe_3_O_4_, Fe_3_O_4_@SiO_2_ and fluorosilane surface modification Fe_3_O_4_@SiO_2_ in photos and tables; Figure S3: Size distribution of of Fe_3_O_4_, Fe_3_O_4_@SiO_2_ and fluorosilane surface modification Fe_3_O_4_@SiO_2_ by dynamic light scattering measurement; Figure S5: Energy-dispersive X-ray spectroscopy (EDS) of Polystyrene micro particles (S5a), magnetic PS micro particle (S5b) and platinum coated magnetic PS micro particles (S5c). Supplemental Video S1: Self-propulsion of the Janus motor in H_2_O_2_; Video S2: Magnetic guidance on the motor's movement; Video S3: Magnetic control on the orientation of the motors.
