# Supplementary material for: Mini-EmulsionFabricated Magnetic and Fluorescent Hybrid Janus Micro-Motors
Source: Micromachines (Basel). 2018 Feb 15;9(2):83. doi: 10.3390/mi9020083 (PMC6187295; doi:10.3390/mi9020083)
Supplement: Supplementary file 1 [file micromachines-09-00083-s001.zip › micromachines-267442-supplementary/supplementary materials.pdf]

# Supplementary Materials: Mini-Emulsion-Fabricated Magnetic and Fluorescent Hybrid Janus Micro-Motors

**Jiapu Jiao, Dandan Xu, Yuhuan Liu, Weiwei Zhao, Jiaheng Zhang, Tingting Zheng, Huanhuan Feng and Xing Ma**

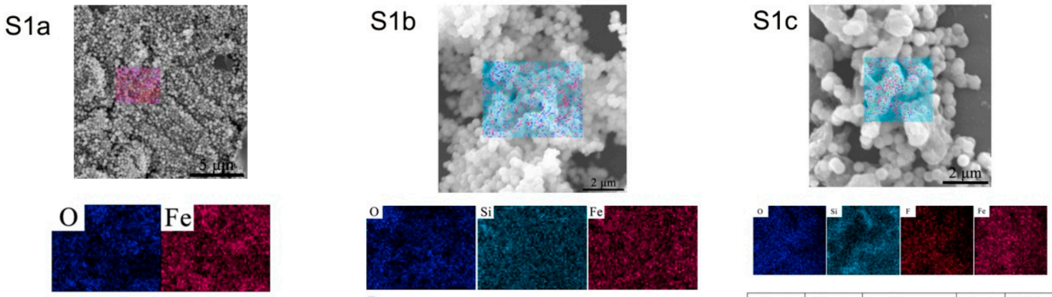


| Element Number | Element Symbol | Element Name | Atomic Conc. | Weight Conc. |
|---|---|---|---|---|
| 8 | O | Oxygen | 69.37 | 39.74 |
| 26 | Fe | Iron | 17.33 | 34.66 |

| Element Number | Element Symbol | Element Name | Atomic Conc. | Weight Conc. |
|---|---|---|---|---|
| 8 | O | Oxygen | 65.05 | 39.39 |
| 26 | Fe | Iron | 12.63 | 26.70 |
| 14 | Si | Silicon | 20.72 | 22.03 |

| Element Number | Element Symbol | Element Name | Atomic Conc. | Weight Conc. |
|---|---|---|---|---|
| 8 | O | Oxygen | 52.02 | 33.30 |
| 26 | Fe | Iron | 9.87 | 22.05 |
| 14 | Si | Silicon | 20.13 | 22.62 |
| 9 | F | Fluorine | 15.66 | 11.91 |

**Figure S1.** Energy-dispersive X-ray spectroscopy (EDS) of $Fe_3O_4$ (**S1a**), $Fe_3O_4@SiO_2$ (**S1b**) and fluorosilane surface modification $Fe_3O_4@SiO_2$ (**S1c**).

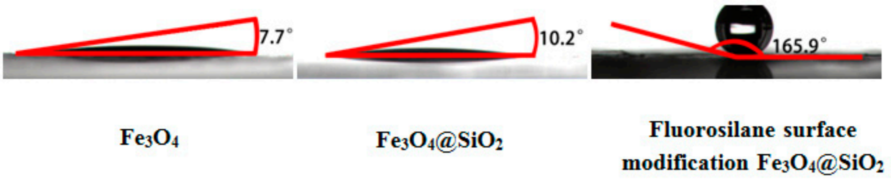


| Contact Angle | $Fe_3O_4$ | $Fe_3O_4@SiO_2$ | Fluorosilane surface modification $Fe_3O_4@SiO_2$ |
|---|---|---|---|
| Average(°) | 8.1 | 10.2 | 170.4 |
| SD(°) | 1.2 | 0.1 | 3.5 |

**Figure S2.** Contact angle measurement of Fe3O4, Fe3O4@SiO2 and fluorosilane surface modification Fe3O4@SiO2 in photos and tables.

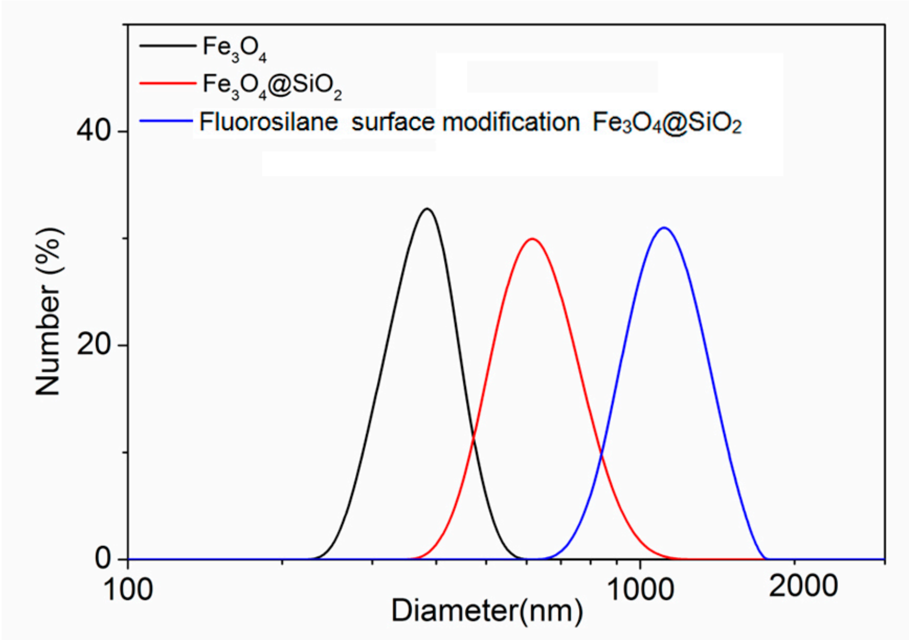


**Figure S3.** Size distribution of of $Fe_3O_4$, $Fe_3O_4@SiO_2$ and fluorosilane surface modification $Fe_3O_4@SiO_2$ by dynamic light scattering measurement.

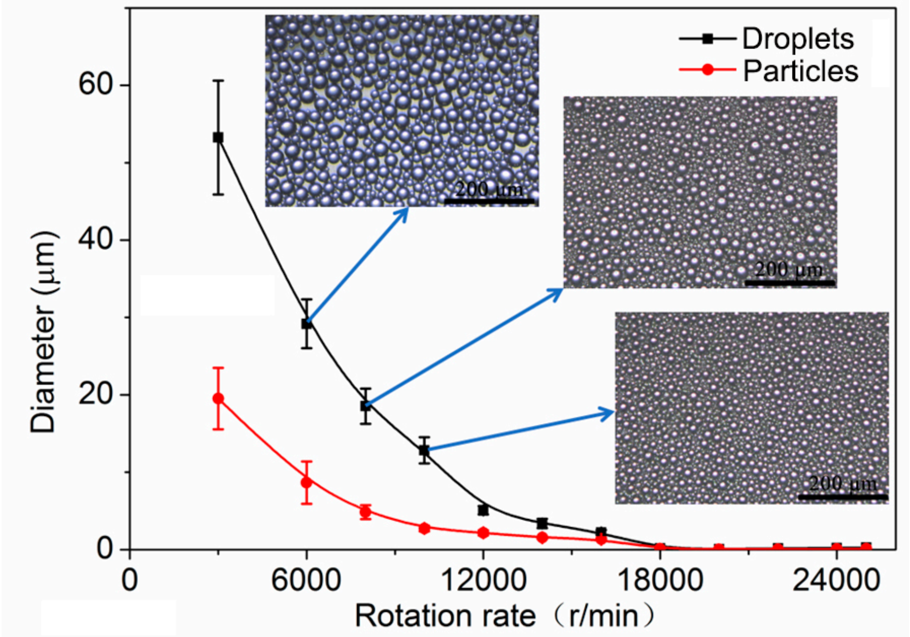


**Figure S4.** The relationship between size and rotation speed.

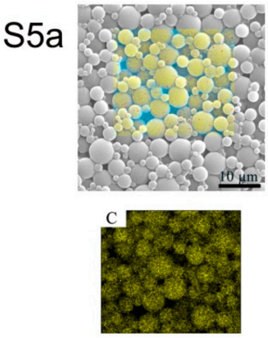


| Element Number | Element Symbol | Element Name | Atomic Conc. | Weight Conc. |
|---|---|---|---|---|
| 6 | C | Carbon | 78.77 | 47.38 |

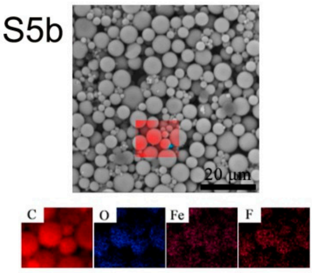


| Element Number | Element Symbol | Element Name | Atomic Conc. | Weight Conc. |
|---|---|---|---|---|
| 6 | C | Carbon | 86.32 | 54.45 |
| 8 | O | Oxygen | 6.84 | 5.75 |
| 26 | Fe | Iron | 0.04 | 0.12 |
| 9 | F | Fluorine | 0.18 | 0.18 |

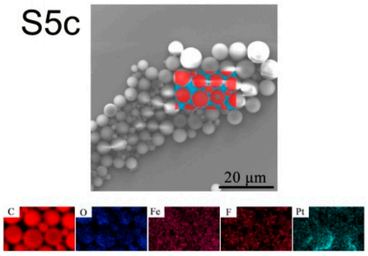


| Element Number | Element Symbol | Element Name | Atomic Conc. | Weight Conc. |
|---|---|---|---|---|
| 6 | C | Carbon | 91.70 | 75.93 |
| 8 | O | Oxygen | 0.62 | 0.68 |
| 26 | Fe | Iron | 0.02 | 0.09 |
| 9 | F | Fluorine | 0.31 | 0.41 |
| 78 | Pt | Platinum | 0.75 | 10.13 |

**Figure S5.** Energy-dispersive X-ray spectroscopy (EDS) of Polystyrene micro particles (**S5a**), magnetic PS micro particle (**S5b**) and platinum coated magnetic PS micro particles (**S5c**).
